# What about N? A methodological study of sample-size reporting in focus group studies

**DOI:** 10.1186/1471-2288-11-26

**Published:** 2011-03-11

**Authors:** Benedicte Carlsen, Claire Glenton

**Affiliations:** 1 Uni Rokkan Centre, Nygårdsgt 5, N-5015 Bergen, Norway; 2 SINTEF Society and Technology, P.O. Box 124 Blindern, Oslo 0314, Norway; 3 The Norwegian Knowledge Centre for the Health Services, P.O. Box 7004 St. Olavs plass, N-0130 Oslo, Norway

## Abstract

**Background:**

Focus group studies are increasingly published in health related journals, but we know little about how researchers use this method, particularly how they determine the number of focus groups to conduct. The methodological literature commonly advises researchers to follow principles of data saturation, although practical advise on how to do this is lacking. Our objectives were firstly, to describe the current status of sample size in focus group studies reported in health journals. Secondly, to assess whether and how researchers explain the number of focus groups they carry out.

**Methods:**

We searched PubMed for studies that had used focus groups and that had been published in open access journals during 2008, and extracted data on the number of focus groups and on any explanation authors gave for this number. We also did a qualitative assessment of the papers with regard to how number of groups was explained and discussed.

**Results:**

We identified 220 papers published in 117 journals. In these papers insufficient reporting of sample sizes was common. The number of focus groups conducted varied greatly (mean 8.4, median 5, range 1 to 96). Thirty seven (17%) studies attempted to explain the number of groups. Six studies referred to rules of thumb in the literature, three stated that they were unable to organize more groups for practical reasons, while 28 studies stated that they had reached a point of saturation. Among those stating that they had reached a point of saturation, several appeared not to have followed principles from grounded theory where data collection and analysis is an iterative process until saturation is reached. Studies with high numbers of focus groups did not offer explanations for number of groups. Too much data as a study weakness was not an issue discussed in any of the reviewed papers.

**Conclusions:**

Based on these findings we suggest that journals adopt more stringent requirements for focus group method reporting. The often poor and inconsistent reporting seen in these studies may also reflect the lack of clear, evidence-based guidance about deciding on sample size. More empirical research is needed to develop focus group methodology.

## Background

Transparency and accountability are key elements in any research report, not least in qualitative studies. Thorough reporting of methods allows readers to assess the quality and relevance of research findings. In addition, for qualitative research methodology to advance, information about how these methods are used and how they work best is needed.

A focus group or focus interview is commonly defined as a method of collecting research data through moderated group discussion based on the participants' perceptions and experience of a topic decided by the researcher [1-6]. Focus groups differ from group interviews in that the emphasis is on the interaction between the participants rather than between the moderator or researcher and the participants [4]. In recent years, focus groups have become increasingly popular within health science research; in a Medline search in 1999, Twohig and Putnam [7] found no focus group studies before 1985 but more than 1000 studies between 1985 and 1999. Focus groups are well suited to explore people's subjective experiences and attitudes, and health researchers are repeatedly encouraged to use focus groups to evaluate health services, to elicit the views of key stakeholders or decision makers or to explore the views of marginalised groups that typically would not respond to a postal survey or would be intimidated by a conventional interview situation [2,5,7,8]. Focus group interviews are also recommended as a pre- or post-study to prepare or interpret data from surveys or trial studies.

Despite this apparent sharp increase in focus group studies in health sciences journals since the 1980 s, there is surprisingly little knowledge about how this method is used and why [1,9,10]. In 1993, one of the "founding fathers" of focus group research methodology, Richard Krueger, complained that the field is burdened with "poor design and shoddy reporting" [11]. After reviewing the field in 1996, Morgan concluded that "At present the reporting of focus group procedures is a haphazard affair at best." Twohig and Putnam's review [7] of focus group studies carried out in the 1990 s showed a "tremendous variation" in how the method was used and concluded that reporting is often inadequate. We were interested in discovering whether there is more reason for optimism a decade later.

There is an extensive literature, ranging from academic classics in grounded theory to pragmatic "how to do" books, that teaches novices how focus groups are usually conducted and indicates how to organise focus groups and how to analyse the data, often within a restricted budget and time span [2,4-6,12-17]. Within this literature, advice on how to decide on the number of groups to conduct is often meagre compared to advice on other aspects of the method. In fact, some teaching books claim that there are no existing guidelines for deciding number of groups [13,16,18].

Most of this guidance recommends that the focus group should be the unit of analysis in focus group-studies. In line with this, sample size should refer to number of groups and not the total number of participants in a study. However, there is of course an intrinsic relationship between these two aspects of sample size. Thus, some authors suggest that if the number of participants in a study is small, it is possible to increase the number of groups by reducing the size of the groups [4]. Guidance on group size is common and seldom goes beyond a minimum of 4 and a maximum of 12 participants per group [2,5,6,13]. Here, it is interesting to note that Fern's experimental study indicated that more information is obtained by conducting two groups of four participants then one group of eight participants [19].

The strength of qualitative methods is their ability to explore the depth and complexity of phenomena. Thus, Sandelowski, in her discussion of sample size in qualitative research, emphasizes that both too few and too many groups can lower the quality of focus group studies [20]. Quantity must be balanced against quality, and the more hours of taped interviews or pages of transcribed material, the less depth and richness the authors will be able to extract from the material [21]. While this knowledge does not directly offer advice regarding how to achieve the optimal number of focus groups, it does highlight an issue that authors should be aware of when determining number of groups.

Most advice about sample size in focus group studies refers to "point of saturation" or "theoretical saturation", The term "theoretical saturation" was introduced by Glaser and Strauss [22] in 1967 in their outline of "grounded theory" and has since spread to most qualitative research environments, albeit in a simplified version, usually referred to as "data saturation". In this approach, interviews should be conducted with different categories of informants following a line of "theoretical sampling" to continuously construct and refine theory or hypotheses. The method requires that data collection, i.e. recruiting, interviewing and analysis, is conducted as an iterative process for each interview, thus representing quite a different approach from the traditional quantitative design of successively calculating sample size beforehand and analysing all data collected [23,24].

The concept of point of saturation has been criticised for being too vague to operationalise [25]. Glaser and Strauss suggest that researchers should: "sample until theoretical saturation of each category is reached" [22](1967:61-62, 11-112) while Strauss and Corbin state that researchers should collect data until "no new or relevant data seem to emerge regarding a category." [15] (1990:188). However, the authors present no definition of "new or relevant data", and give no advice regarding the number of interviews with no new information that is required before the researcher can be reasonably certain that saturation has been reached.

More pragmatic authorities on focus group methodology recognise that many researchers work on assignment and their funders may require that the number of groups are decided pre-study [2,4,12]. The recommendations and references to what is common regarding number of groups vary within these text books. In general they recommend from two to five groups per category of participants. However, the authors usually underline that these are only rules of thumb and that the number of focus groups depends on the complexity of the research question and the composition of the groups. Often, the advice given is to follow the rules of thumb but to suggest a slightly higher number to be "on the safe side". Some authors suggest that the researchers then use point of saturation as they go along to decide the final number of groups needed [2,4].

While some of these text books still refer to Glaser and Strauss, others do not. In these cases the concept of theoretical sampling and theoretical saturation and the theory behind it seem to have been lost on the way and replaced with the less theoretically sustained terms of "purposive sampling" and "data saturation" or merely "saturation". However, the basic procedure of selecting informants and deciding on the number of groups through a constant process of analysing data and obtaining new data until no new essential information is found, remains.

It has been claimed by critics within the field of qualitative methodology that the concept of saturation is often misused in research reports [23,26]. Charmaz [25] (2005:528) claims that "Often, researchers invoke the criterion of saturation to justify small samples - very small samples with thin data." In reality, these small sample sizes may, in fact, be a result of lack of time or lack of funds. Whether "saturation" has thus become the magic word which obscures faulty designs in focus group reports, as Charmaz insinuates, has yet to be investigated.

In general, empirical research into how focus group methods are used and work is scarce. We have found only two review studies that document and discuss sample size in focus group studies [7,8] and to our knowledge no study has yet assessed how decisions about sample size in focus group studies are reported. Nor does the effectiveness of different sample sizes appear to have been evaluated. This lack of empirical evidence suggests that advice offered with regard to sample size is, as a rule, based on common assumptions or personal experience with the method.

## Objectives

Our objective is twofold. First, we aim to survey sample size in current focus group studies in health science journals. Second, we aim to survey and assess the extent to which researchers describe and justify the number of focus groups they carry out.

## Methods

We carried out a structured search for papers that included focus group methods, and extracted and analysed quantitative and qualitative information about the authors' use of these methods.

### Eligibility criteria

We searched PubMed Central in 2009 for primary studies that used focus groups and were published in 2008. We chose PubMed Central because it offers good coverage of medical journals and allowed us to apply a "Free Full Text filter" to limit searches to publications from open-access journals. This approach gave us a manageable sample of studies and easy and immediate access to papers, and allows others to easily access publications cited in this review and check our conclusions.

As mentioned, definitions of focus group interviews usually refer to researcher-initiated gathering of a small group of people with the aim of facilitating discussion about a given topic [1,4-6]. However, we included any study where the authors themselves used the term "focus group" to refer to their method of data collection. We included studies using focus groups only and studies using mixed methods. We chose studies published in 2008 as we started working with the review in 2009 and wanted to review the state of the art in the field. In addition we repeated the same search for 2003 and 1998, i.e. five and ten years earlier as a crude check on the expansion of the field.

We excluded papers that described planned studies and papers that described internet-based focus groups as we assumed that recruiting participants for such groups possibly invokes other challenges than recruiting participants to face-to-face groups.

### Search methods for identification of studies

We searched Pubmed Central using the following search string:

"2008"[Publication Date] AND ("focus group"[Title/Abstract] OR "focus groups"[Title/Abstract] OR "focus interviews"[Title/Abstract]) AND (English[lang] AND pubmed pmc local[sb]). The search was conducted on December 21^st ^2009.

### Data selection, extraction and analysis

One review author (BC) independently assessed the relevance of full text versions of all papers identified from the electronic searches. Decisions to exclude papers were checked by the other review author (CG).

Both authors independently extracted data from the first ten included studies using a standard form based on the variables we aimed at analysing. The data were then checked against each other, and, if necessary, reference was made to the original paper. Any discrepancies between the two data extraction sheets were discussed by the two reviewers and resolved by consensus. For the remaining studies, one reviewer (BC) extracted the data alone and checked with the other author when in doubt.

We extracted data about the following aspects as numerical data:

1. Number of focus groups in each study

2. Maximum and minimum number of participants in the focus groups in each study

3. Total number of participants

4. Whether any explanation for number of groups was given

5. Whether this explanation was tied to:

◦ Practical issues (such as convenience when recruiting or limited resources to conduct interviews)

◦ Recommendations in the literature (pragmatic guidelines to number of focus groups)

◦ Saturation

◦ Capacity when analyzing, i.e. balancing between depth and breadth of data

We analysed this data descriptively (with SPSS 15.0).

In addition, we carried out a matrix-based qualitative analysis of the texts. Here, we explored the study reports adopting the same approach that we would have used for transcribed interview material, using a cross-case thematic analysis described by Miles and Huberman, among others [27]. Specifically, we extracted and condensed relevant passages from all included papers in a separate document with our comments regarding level and style of reporting and the authors' explanations for sample size. We especially focussed on:

• How clear and accessible information regarding sample size was in each study

• Whether explanations for number of focus groups was clear, credible and consistently reported

• Whether and how the number of groups were referred to by the authors when discussing study strengths and limitations

## Results

### The current status of sample size in focus group studies

Through our electronic database search we identified 240 papers published in 2008. In comparison, our search for 1998 and 2003 resulted in 23 and 62 studies respectively.

We considered the full text version of each of the 240 papers from 2008 for inclusion in the review. Twenty papers were excluded after reading the full text version. Of these, six were excluded because they were planned studies, four were excluded because the focus groups were Internet-based, three were excluded because they were not primary studies and seven were excluded because they did not, in fact, report the results of focus group studies even though the term "focus groups" appeared in their abstracts. (Some of these referred to focus groups as part of an intervention that was evaluated in the reported study).

Two hundred and twenty papers met our inclusion criteria. (See Additional file 1 for a list of included studies.) These were published in 117 different journals. Sixty-one percent had used mixed methods of data collection, while 39% had used focus groups only.

Many of the 220 papers were characterised by an insufficient reporting of sample size:

• 22 (10%) did not report number of focus groups

• 42 (19%) did not report total number of participants

• 102 (46%) did not report minimum and maximum number of participants in the focus groups

In addition, it was sometimes difficult for us to find focus group and participant numbers as this information was sometimes reported in different sections of the papers (See for example [28,29]. For mixed method studies, it was sometimes difficult to separate between sample size information for different data collection methods (See for example [30]).

Those papers that did report numbers of groups and participants showed a great range in these numbers, but data distribution was positively skewed, i.e. there were many studies with a few focus groups and few studies with high numbers of focus groups (Table 1).

**Table 1:** Overview over sample sizes in the included studies.

		Number of groups	Number of participants	Min number in group	Max number in group
N	Valid	198	178	118	119
	Missing	22	42	102	101
Mean		8.4	45.5	5.2	8.7
Median		5	32	5	8
Range		1 to 96	3 to 279	1 to 13	3 to 20

### Authors' explanations for number of focus groups

Authors' explanations of how they had decided on or ended up with the number of focus groups carried out varied, but were often unclear or completely lacking (e.g. [31,32]). (See Table 2.) When authors used mixed methods, explanations of sample size for the quantitative part of the study were often meticulous, while the sample size of the focus interviews in contrast was often unclear and superficial, as in this example:

**Table 2:** Crosstabulation; Number of groups and authors' explanation for number of groups

Number of groups	Authors' explanation					Total
	
	No explanation	Practical reasons	Recommended by literature	Saturation	Capacity to analyse data	
1	11	0	0	0	0	11
2	17	0	1	0	0	18
3	24	0	2	3	0	29
4	25	0	3	5	0	33
5	7	2	0	4	0	13
6	14	0	0	5	0	19
7	8	0	0	1	0	9
8	12	1	0	4	0	17
9	2	0	0	4	0	6
10	4	0	0	0	0	4
11	3	0	0	0	0	3
12	6	0	0	0	0	6
13	1	0	0	2	0	3
14-96	27	0	0	0	0	27
No info	22	0	0	0	0	22

**Total**	**183**	**3**	**6**	**28**	**0**	**220**

*- purposive sampling was undertaken till the necessary number was attained *[33].

Of the 220 studies included, 183 (83,2%) gave no explanation for number of focus groups (i.e. 37 did give some type of explanation). Often, authors explained the number of participants rather than the number of focus groups [34,35]. Typical for these cases were situations where focus group studies were carried out alongside clinical intervention studies and where the authors had invited all participants from the intervention study to participate in the focus group interviews [34,36]. As these accounts explain the number of participants rather than the number of focus groups, this was categorised as "no explanation".

From Table 2 we also see that no study justified the number of groups by referring to the need to balance data quality and quantity (capacity to analyse data). The table also shows that all the explanations for sample size were found in studies that had between two and 13 focus groups. We tested if there was any correlation between the presence of an explanation for number of focus groups and the number of groups conducted (Pearson correlation). We found no linear relationship. None of the eleven single-group studies attempted to justify why one group was sufficient. These studies all use mixed methods and the qualitative assessment showed that they typically used the focus group study as a pilot for developing questionnaires etc.

As noted, none of the studies that included more than 13 groups gave any explanations for sample size and the qualitative assessment showed that none of the studies referred to a high number of groups as a study limitation. On the contrary, when authors considered their sample size to be relatively high, this was seen as advantageous:

*By conducting a large number of focus groups with a significant cross-section of the facility's employees, we reduced the possibility that results would be dramatically affected by a single focus group or methodological choice during coding *[37].

On the other hand, when authors had included only a small number of focus groups, they frequently described this as a limitation. However, in several of these studies, authors also claimed the number of focus groups had been determined by data saturation. This should, in theory, imply that the number of focus groups was, in fact, appropriate. (See for example [38-43]). But while authors often described a small number of focus groups as a weakness, several studies justified their choice of method with reference to the possibilities that focus groups gave to go in depth and provide a thick description of the issue.

### Saturation

Among the 37 studies that did give an explanation for the number of focus groups, 28 claimed that they had stopped once they had reached a point of saturation. However, more than half of these explanations (15 of 28) did not report convincingly that an iterative process, involving data collection and analysis that ended with saturation, had taken place. The reason for this was mainly inconsistencies in the description of the methodological procedures. One common example was that, despite their reference to point of saturation, their number of focus groups was pre-determined, as the extraction below shows (See also [44,45]):

*Three focus groups were conducted with residents of a specific community. It was a small community, and it was determined that after three focus groups, there would be a saturation point regarding their barriers and suggested solutions *[46].

Other common examples where claims of saturation appeared unsubstantiated were studies that used convenience sampling, and included everyone who volunteered to participate, instead of purposive or theoretical sampling, and where, in addition all data seemed to have been gathered before the analysis (See for example [41,42,47-49]).

We also noted that no author reported or discussed the number of focus groups that had been conducted with no new relevant information before it was decided that a point of saturation had been reached. On the contrary, authors normally gave superficial and vague references to the process of reaching point of saturation, many of them referring to a "feeling" of having reached this point:

*Focus groups were [...] conducted until we felt "saturation" (no new themes identified) was reached *[41].

*At the end of the fifth focus group, we felt that theme saturation had been met, so we stopped recruitment... *[50]

We also found some examples of adequate reporting regarding point of saturation. For example Barimani et al [51] explained their use of grounded theory and described how a constant comparison between empirical findings and theory had guided their sampling procedure until theoretical saturation was met. This paper was, however, unusually long, around 9000 words. Another example is Shuval et al [52]. In their paper of around 3000 words, the authors have not specifically referred to the term "saturation" but have offered a clear description of the sampling procedure and how saturation was met:

*We used purposeful sampling *[with reference to Patton 1990] *to select key informants (on the basis of researchers' acquaintance), promote group interaction, and capture the diverse characteristics of participants (i.e., sex, age, living environment, ethnicity/religion, and self-reported income). All students whom we approached agreed to participate in focus groups*.

[...]

*Focus groups were held until no new themes emerged (9). [...] Researchers reviewed the transcripts to reflect on each session before conducting the next, thereby enabling newly identified concepts to be examined in subsequent sessions *[52].

### Recommendations in the literature

Six of the 37 studies that gave an explicit explanation for the number of focus groups referred to rules of thumb in the literature. These were mostly references to pragmatic guidelines of how many groups are necessary to reach a point of saturation (e.g. [53]). As the example below illustrates, authors reported that the field lacks consistent guidelines.

*Recommendations for both the number of focus groups and sample size vary. The number of recommended sessions depends on the complexity of the study design and the target sample's level of distinctiveness *[18-21]*. Stewart et al. (2007) observed that rarely are more than 3-4 focus groups conducted in the social sciences. We felt that two groups at each site would limit bias that might be seen in a single group or site and allow us to examine themes common across groups *[54].

However, when we checked with the literature, referrals to recommendations were not always accurate. For example Gutterling et al, conducted three focus groups and explain the number thus:

*For good results, just a few focus groups are sufficient, as data become saturated and little new information emerges after the first few groups [Morgan 96] *[55].

Looking up Morgan [1], we found that he claims that most studies use four to six groups because they then reach saturation, but he also underlines that the more categories of participants and less standardisation of questions, the higher number of focus groups.

### Practical reasons

Three of the 37 studies reported practical reasons for the number of focus groups conducted. The information offered regarding recruitment constraints was incomplete as, for example, in this explanation:

*The number of available participants was limited and the number of focus groups was therefore few *[56].

Two of the explanations appeared to be tied to difficulties in recruiting participants to additional groups [56,57], while one mentioned limited resources ("budgetary and staffing constraints") which led the researchers to decide pre-study to conduct five focus groups [38]. This last study also claimed to have reached saturation (without stating this as the procedure for deciding number of groups):

*However, data analysis indicates that all themes reached saturation, meaning additional participants would likely not have added to the depth or breadth of parent responses *[38]

An additional eight studies also described recruitment limitations, but only as an explanation for the total number of participants, not for how many groups the participants were divided into. In these studies, the size of the groups seems to have been decided beforehand, due to text book recommendations, and thus the number of groups was given by the total number of already recruited participants divided by the number of participants per group.

## Discussion

The results from our searches from 1998, 2003 and 2008 support the claims that there has been an increase in focus group studies over the last ten years. The wide range of health journals publishing focus group studies in 2008 indicates that this method is now widely accepted. At the same time, the fact that many journals publish only one or two focus group studies a year could also mean that the methodological competence among editors and reviewers to assess focus group studies is lacking.

The great variation in the number of focus groups that we registered was surprising, and was wider than authors of teaching materials and text books assume. For example Stewart et al [13] (2007:58) claim: "Most focus group applications involve more than one group, but seldom more than three or four groups." Twohig and Putnam also found a much narrower variation in their review of focus group studies in primary care research, with a range of two to eight groups per study [7].

Overall, reporting of sample size and explanations for this size was poor. Where such explanations were given, our study confirms the dominant role of the concept of data saturation.

We also discovered that all explanations were found in studies of between two and 13 groups. Some of these studies refer to existing pragmatic guidelines to justify their numbers, although the two to five focus groups per category recommended in these guidelines sometimes appear to have become two to five groups in total in the studies. We could speculate that studies using only one focus group, a number that goes against the rules of thumb offered by these guidelines, is simply too hard to justify and explanations are therefore evaded. Also, all the single group studies were mixed methods studies, where the focus group typically was used as a pilot to develop or test a questionnaire for the survey part of the study. In these examples, it is understandable that the focus group is offered less attention than the main part of the study. At the other end of the scale, one could also speculate that when the sample size reaches two-digit numbers, a "quantitative study logic" kicks in where a big N is seen as a positive asset and therefore less important to justify.

Roughly half of the studies that referred to data saturation as an explanation for number of focus groups did not appear to be consistent in their use of approach. These findings support earlier reviews of the field. Twohig and Putnam [7], were also startled by the variation in procedures and reporting of focus group studies, and Webb and Kevern [58], who reviewed focus group studies in nursing research, found that authors used terms such as "Grounded theory" in non-rigorous ways. These authors also conclude that researchers, on the whole, did not follow basic premises for reaching saturation such as concurrent data generation and analysis, or an "iterative process" [15]. Our study shows the same tendencies, and suggests that the increased use of focus groups in health care studies has not led to an improvement in the quality of reporting.

We were also struck by what we did not find. In our own experience with focus group research, recruitment problems are much more common than this review indicates. In addition, a number of practical limitations arise that can limit the number of focus groups conducted, including limited money and time. Excepting one study, where resource constraints were brought up, the only practical limitations mentioned were difficulties in recruiting more participants. Another non-finding was that none of the studies discussed a large number of focus groups as a potential limitation of the study. Given frequent references in these studies to the advantages of qualitative methodology for eliciting richness and depth of the data, it is not evident why the authors never used the argument that data from a large number of focus groups is difficult to analyse thoroughly.

### How can poor reporting be explained?

The inadequate reporting indicated in our study could reflect the fact that most health science journals do not require specific standards of reporting from contributors presenting qualitative research. However, the poor reporting among these authors also seem to indicate confusion about when and how to decide the number of focus groups, which may reflect a lack of properly described, consistent advice to researchers wishing to carry out focus group-based data collection.

The lack of attention to sample size in the teaching material could easily be perceived as an indication that sample size is unimportant in such studies. In addition, the advice that is offered is confusing and sometimes conflicting. While the Glaser and Strauss's procedure of theoretical saturation instructs authors to use theoretical sampling and to analyse and collect data iteratively until saturation is achieved, it does not offer a detailed interpretation of how to operationalise this approach. The "how to do" literature on focus group methodology, on the other hand, offers pragmatic advice regarding the number of groups that researchers should expect to conduct before point of saturation is reached, but do not clarify how to decide about point of saturation in practice. This advice may thus tempt researchers to follow their suggestions for number of groups and do the analysis after collecting all data. Then, as they expect data to be saturated, their critical sense could be undermined when drawing conclusions about saturation.

Despite problems associated with the practical application of the concept of data saturation, it seems to have become something of an ideal in qualitative health research. This could be due to the fact that it is the only theory that offers advice, albeit, poorly operationalised, about the exact number of interviews needed. It is plausible that editors in the traditionally positivistic realm of health research are inclined to prefer explanations for exact number of groups. Practical limitations might not be as acceptable, especially not explicit references to economic or resource limitations.

In the methodology discussions of these studies, authors often point out that small sample sizes are legitimate in qualitative studies. At the same time, they often feel the need to justify small sample sizes which they invariably see as a study limitation. This may be a consequence of the fact that qualitative studies are still in a minority in health science journals. Here, more positivistic traditions may make it difficult to argue that a qualitative study can have too many groups. Nevertheless, the quality of qualitative studies does depend on the depth and richness of the data and its analysis. Reference to the trade-off between number of focus groups and the thickness of our description should therefore be an acceptable explanation for (a limited) sample size. There is also an ethical side to sample size: an excessive number of interviews means placing a burden on patients or health workers that is not legitimised by added scientific value and can thereby be seen as unethical.

### Study strengths and limitations

A limitation of our study was that our sample was taken from open-access journals. While this gave us easy and immediate access to a manageable sample of studies and also allows our readers to easily check our results, a quick search without the open access filter indicates that our sample represents less than 20% of all published focus group studies in 2008. We know relatively little about how our sample might differ from the remaining 80% of available studies. Current research does suggest that articles published in open-access journals are more often cited than other articles [59]. It is therefore possible that the articles we evaluated are of a higher profile than other non-open-access articles and may be more likely to serve as examples for other researchers. While we emphasise that our findings are primarily valid for open access studies, it therefore seems all the more important to secure the quality of these reports.

We decided to include all studies claiming to be focus group studies. We have therefore also included mixed method studies, where the focus group interviews are often part of a predominantly quantitative design. In such studies the authors may not aim to adhere to standards for reporting qualitative studies. On the other hand, it could be argued that researchers who report that they have used focus groups should adhere to the methodological standards for such studies.

There are some uncertainties in our findings due to the poor reporting in the material. For instance, it was sometimes difficult to decide whether an explanation for number of focus groups had been given and what this explanation was. The number of studies that give practical reasons for their number of groups or that refers to data saturation is therefore slightly uncertain. Because of meagre and unclear reporting, it was also difficult to tell whether the studies that claimed to have decided on sample size through an iterative research process and point of saturation did, in fact, analyse their data after data collection had ended. Usually, several confusing aspects of the reporting appeared in the same studies.

As our study looked at focus groups only, future research should consider whether sample size reporting of individual interviews shows similar problems.

## Conclusions

While researchers should always provide correct and detailed information about the methods used, our study shows poor and inconsistent reporting of focus group sample size. Editorial teams should be encouraged to use guidelines for reporting of methods for qualitative studies, such as RATS http://www.biomedcentral.com/info/ifora/rats.

Our study also indicates that poor reporting could reflect a lack of clear, evidence-based guidance about how to achieve optimal sample size. To amend this situation, text books and teaching material based on empirical studies into the use of focus group methodology and applicable and precise recommendations are needed. Ironically, one barrier to high-quality methodological studies is the current lack of proper reporting by authors of primary studies.

## Competing interests

The authors declare that they have no competing interests.

## Authors' contributions

BC designed the study, participated in developing the search strategy, conducted the search, extracted relevant data for analysis, participated in analysing the data and drafted the manuscript. CG participated in developing the search strategy, checked extracted data, participated in analysing the data and revised the manuscript AB. Both authors read and rewrote the manuscript and have approved the final manuscript.

## Authors' information

BC and CG are social anthropologists with PhDs in health services research. Both authors have conducted multimethod research, including focus group studies, partly on assignment and have mainly published in health science journals.

## Pre-publication history

The pre-publication history for this paper can be accessed here:

http://www.biomedcentral.com/1471-2288/11/26/prepub

## Supplementary Material

Additional file 1 **The table shows author, journal, number of focus groups and explanation for number of focus groups for all 220 included studies**. Click here for file

## References

[B1] Morgan DL Focus Groups Annual Review of Sociology 1996 22 129 152 10.1146/annurev.soc.22.1.129

[B2] Krueger RA Casey MA Focus groups: a practical guide for applied research 2009 4 Thousand Oaks, California: Sage

[B3] Powell RA Single HM Focus groups Int J Qual Health Care 1996 8 499 504 9117204 10.1093/intqhc/8.5.499

[B4] Morgan DL Focus Groups as Qualitative Research 1997 2 Thousand Oaks: Sage Publications

[B5] Kitzinger J Qualitative Research: Introducing focus groups BMJ 1995 311 299 302 7633241 10.1136/bmj.311.7000.299 PMC2550365

[B6] Bender DE Ewbank D The focus group as a tool for health research: issues in design and analysis Health Transition Review 1994 4 63 79 10147164

[B7] Twohig PL Putnam W Group interviews in primary care research: advancing the state of the art or ritualized research? Fam Pract 2002 19 278 284 10.1093/fampra/19.3.278 11978719

[B8] Halcomb EJ Gholizadeh L DiGiacomo M Phillips J Davidson PM Literature review: considerations in undertaking focus group research with culturally and linguistically diverse groups Journal of Clinical Nursing 2007 16 1000 1011 10.1111/j.1365-2702.2006.01760.x 17518876

[B9] Dyas J Apekey T Tilling M Siriwardena AN Strategies for improving patient recruitment to focus groups in primary care: a case study reflective paper using an analytical framework BMC Medical Research Methodology 2009 9 65 10.1186/1471-2288-9-65 19772603 PMC2759948

[B10] Fern EF Focus groups: A review of some contradictory evidence, implications, and suggestions for future research Advances in consumer research 1983 10 121 126

[B11] Krueger RA Morgan DL Quality control in focus group research Successful Focus Groups: Advancing the State of the Art 1993 Newbury Park: Sage 65 85

[B12] Hughes D DuMont K Using focus groups to facilitate culturally anchored research American Journal of Community Psychology 1993 21 775 806 10.1007/BF00942247 8085565

[B13] Stewart DW Shamdasani PN Rook DW Focus Groups. Theory and Practice 2007 Thousand Oaks: Sage Publications

[B14] Bryant A Charmaz K The SAGE Handbook of Grounded Theory 2007 London: SAGE

[B15] Strauss A Corbin J Basics of Qualitative Research. Grounded Theory Procedures and Techniques 1990 Newbury Park, California: SAGE Publications

[B16] Bowling A Research methods in health 2002 2 Berkshire, UK: Open University Press

[B17] Morgan DL The Focus Group Guide Book 1998 Thousand Oaks: SAGE Publications

[B18] Patton MQ Qualitative Research and Evaluation Methods 2002 London: Sage Publications

[B19] Fern EF The Use of Focus Groups for Idea Generation: The Effects of Group Size, Acquaintanceship, and Moderator on Response Quantity and Quality Journal of Marketing Research 1982 19 1 13 10.2307/3151525

[B20] Sandelowski M Sample-size in qualitative research Research in Nursing & Health 1995 18 179 183 10.1002/nur.4770180211 7899572

[B21] Morse JM Bryant A, Charmaz K Sampling in Grounded Theory The SAGE Handbook of Grounded Theory 2007 London: SAGE Publications

[B22] Glaser BG Strauss AL The Discovery of Grounded Theory: Strategies for Qualitative Research 1967 Chicago: Aldine

[B23] Denzin NK Lincoln YS (Eds) The SAGE Handbook of Qualitative Research 2005 Third Thousand Oaks

[B24] Kennedy TJT Lingard LA Making sense of grounded theory in medical education Medical Education 2006 40 101 108 10.1111/j.1365-2929.2005.02378.x 16451236

[B25] Charmaz K Denzin NK, Lincoln YS Grounded Theory in the 21st Century: Applications for Advancing Social Justice Studies The SAGE Handbook of Qualitative Research 2005 Third London: SAGE Publications 507 535

[B26] Charmaz K Mitchell RG Atkinson P, Coffey A, Delamont S, Lofland J, Lofland L Grounded Theory in Ethnography Handbook of Ethnography 2001 Los Angeles: SAGE

[B27] Miles M Huberman A Qualitative data analysis: an expanded sourcebook 1994 London: Sage

[B28] Constantine R Brownstein JN Hoover S Wordlaw-Stinson L Orenstein D Jones P Farris R Strategies for controlling blood pressure among low-income populations in Georgia Prev Chronic Dis 2008 5 A52 18341787 PMC2396988

[B29] Cabassa LJ Hansen MC Palinkas LA Ell K Azucar y nervios: explanatory models and treatment experiences of Hispanics with diabetes and depression Soc Sci Med 2008 66 2413 2424 10.1016/j.socscimed.2008.01.054 18339466 PMC2475593

[B30] Kothari A Driedger SM Bickford J Morrison J Sawada M Graham ID Crighton E Mapping as a knowledge translation tool for Ontario Early Years Centres: views from data analysts and managers Implement Sci 2008 3 4 10.1186/1748-5908-3-4 18205913 PMC2265301

[B31] Winickoff JP Park ER Hipple BJ Berkowitz A Vieira C Friebely J Healey EA Rigotti NA Clinical effort against secondhand smoke exposure: development of framework and intervention Pediatrics 2008 122 e363 375 10.1542/peds.2008-0478 18676523 PMC2774730

[B32] Lloyd CE Johnson MR Mughal S Sturt JA Collins GS Roy T Bibi R Barnett AH Securing recruitment and obtaining informed consent in minority ethnic groups in the UK BMC Health Serv Res 2008 8 68 10.1186/1472-6963-8-68 18373876 PMC2311303

[B33] Perez F Aung KD Ndoro T Engelsmann B Dabis F Participation of traditional birth attendants in prevention of mother-to-child transmission of HIV services in two rural districts in Zimbabwe: a feasibility study BMC Public Health 2008 8 401 10.1186/1471-2458-8-401 19061506 PMC2612666

[B34] Wild K Boise L Lundell J Foucek A Unobtrusive In-Home Monitoring of Cognitive and Physical Health: Reactions and Perceptions of Older Adults J Appl Gerontol 2008 27 181 200 10.1177/0733464807311435 19165352 PMC2629437

[B35] Tentler A Silberman J Paterniti DA Kravitz RL Epstein RM Factors affecting physicians' responses to patients' requests for antidepressants: focus group study J Gen Intern Med 2008 23 51 57 10.1007/s11606-007-0441-8 17987348 PMC2173928

[B36] Kerrison S Laws S Cane M Thompson A The patient's experience of being a human subject J R Soc Med 2008 101 416 422 10.1258/jrsm.2007.070288 18687865 PMC2500238

[B37] Rosen T Lachs MS Bharucha AJ Stevens SM Teresi JA Nebres F Pillemer K Resident-to-resident aggression in long-term care facilities: insights from focus groups of nursing home residents and staff J Am Geriatr Soc 2008 56 1398 1408 10.1111/j.1532-5415.2008.01808.x 18637979 PMC2755096

[B38] Davis AM James RL Curtis MR Felts SM Daley CM Pediatric obesity attitudes, services, and information among rural parents: a qualitative study Obesity (Silver Spring) 2008 16 2133 2140 10.1038/oby.2008.312 18551114 PMC2701507

[B39] Hatchette JE McGrath PJ Murray M Finley GA The role of peer communication in the socialization of adolescents' pain experiences: a qualitative investigation BMC Pediatr 2008 8 2 10.1186/1471-2431-8-2 18190716 PMC2254410

[B40] Burnet DL Plaut AJ Ossowski K Ahmad A Quinn MT Radovick S Gorawara-Bhat R Chin MH Community and family perspectives on addressing overweight in urban, African-American youth J Gen Intern Med 2008 23 175 179 10.1007/s11606-007-0469-9 18071829 PMC2359171

[B41] Power JD Badley EM French MR Wall AJ Hawker GA Fatigue in osteoarthritis: a qualitative study BMC Musculoskelet Disord 2008 9 63 10.1186/1471-2474-9-63 18452607 PMC2386135

[B42] Hsu C Phillips WR Sherman KJ Hawkes R Cherkin DC Healing in primary care: a vision shared by patients, physicians, nurses, and clinical staff Ann Fam Med 2008 6 307 314 10.1370/afm.838 18626030 PMC2478495

[B43] Agampodi SB Agampodi TC Ukd P Adolescents perception of reproductive health care services in Sri Lanka BMC Health Serv Res 2008 8 98 10.1186/1472-6963-8-98 18454869 PMC2386785

[B44] Goldstein NE Mehta D Siddiqui S Teitelbaum E Zeidman J Singson M Pe E Bradley EH Morrison RS "That's like an act of suicide" patients' attitudes toward deactivation of implantable defibrillators J Gen Intern Med 2008 23 Suppl 1 7 12 10.1007/s11606-007-0239-8 18095037 PMC2150628

[B45] Mohler MJ Coons SJ Hornbrook MC Herrinton LJ Wendel CS Grant M Krouse RS The health-related quality of life in long-term colorectal cancer survivors study: objectives, methods and patient sample Curr Med Res Opin 2008 24 2059 2070 10.1185/03007990802118360 18544186 PMC2575021

[B46] Griffin SF Wilson DK Wilcox S Buck J Ainsworth BE Physical activity influences in a disadvantaged African American community and the communities' proposed solutions Health Promot Pract 2008 9 180 190 10.1177/1524839906296011 17728204 PMC2656764

[B47] O'Donnell CA Higgins M Chauhan R Mullen K Asylum seekers' expectations of and trust in general practice: a qualitative study Br J Gen Pract 2008 58 e1 11 10.3399/bjgp08X376104 PMC2593560 19068152

[B48] Joos S Musselmann B Miksch A Rosemann T Szecsenyi J The role of complementary and alternative medicine (CAM) in Germany - A focus group study of GPs BMC Health Services Research 2008 8 127 10.1186/1472-6963-8-127 18549476 PMC2442431

[B49] Neergaard M Olesen F Jensen A Sondergaard J Palliative care for cancer patients in a primary health care setting: Bereaved relatives' experience, a qualitative group interview study BMC Palliative Care 2008 7 1 10.1186/1472-684X-7-1 18197982 PMC2254378

[B50] Nicolaidis C Gregg J Galian H McFarland B Curry M Gerrity M "You always end up feeling like you're some hypochondriac": intimate partner violence survivors' experiences addressing depression and pain J Gen Intern Med 2008 23 1157 1163 10.1007/s11606-008-0606-0 18443884 PMC2517952

[B51] Bariman M Hylander I Linkage in the chain of care: a grounded theory of professional cooperation between antenatal care, postpartum care and child health care Int J Integr Care 2008 8 10.5334/ijic.254 PMC2638018 19209242

[B52] Shuval K Weissblueth E Brezis M Araida A Faridi Z Ali A Katz DL The Role of Culture, Environment, and Religion in the Promotion of Physical Activity Among Arab Israelis Prev Chronic Dis 2008 8 PMC2483551 18558038

[B53] Blixen CE Webster NJ Hund AJ Perzynski AT Kanuch SW Stoller EP McCormick RA Dawson NV Communicating about alcohol consumption to nonharmful drinkers with hepatitis C: patient and provider perspectives J Gen Intern Med 2008 23 242 247 10.1007/s11606-007-0483-y 18172739 PMC2359467

[B54] Castel LD Williams KA Bosworth HB Eisen SV Hahn EA Irwin DE Kelly MA Morse J Stover A DeWalt DA DeVellis RF Content validity in the PROMIS social-health domain: a qualitative analysis of focus-group data Qual Life Res 2008 17 737 749 10.1007/s11136-008-9352-3 18478368 PMC2757448

[B55] Gutteling JJ de Man RA Busschbach JJ Darlington AS Quality of health care and patient satisfaction in liver disease: the development and preliminary results of the QUOTE-Liver questionnaire BMC Gastroenterol 2008 8 25 10.1186/1471-230X-8-25 18570638 PMC2453127

[B56] Nilsson C Skar L Soderberg S Swedish district nurses' attitudes to implement information and communication technology in home nursing Open Nurs J 2008 2 68 72 10.2174/1874434600802010068 19319223 PMC2600856

[B57] Watson DB Thomson RG Murtagh MJ Professional centred shared decision making: patient decision aids in practice in primary care BMC Health Serv Res 2008 8 5 10.1186/1472-6963-8-5 18190683 PMC2248564

[B58] Webb C Kevern J Focus groups as a research method: a critique of some aspects of their use in nursing research Journal of Advanced Nursing 2001 33 798 805 10.1046/j.1365-2648.2001.01720.x 11298218

[B59] Wagner AB Open Access Citation Advantage: An Annotated Bibliography Issues in Science and Technology Librarianship 2010 60 http://www.istl.org/10-winter/article2.html

