# Supplementary material for: What about N? A methodological study of sample-size reporting in focus group studies
Source: BMC Med Res Methodol. 2011 Mar 11;11:26. doi: 10.1186/1471-2288-11-26 (PMC3061958; doi:10.1186/1471-2288-11-26)
Supplement: Additional file 1 — The table shows author, journal, number of focus groups and explanation for number of focus groups for all 220 included studies. [file 1471-2288-11-26-S1.DOC]

| **First author** | **Journal** | **Number of groups** | **Explanation for number of groups** |
| --- | --- | --- | --- |
| Agampodi | BMC Health Serv Res | 4 | No |
| Ahldén | Journal of perinatal Education | 4 | No |
| Ajayi | BMC Health Serv Res | 24 | No |
| Ajuwon | BMC Med Ethics | 1 | No |
| Anderson | Diabetes Care | 5 | No |
| Arnold | Patient Educ Couns | 6 | No |
| Atuyambe | Afr Health Sci | 20 | No |
| Bajcar | Can Fam Physician | Information missing | No |
| Barimah | BMC Complement Altern Med | 3 | No |
| Barimani | Int Journ Integr Care | 5 | Yes |
| Barnes | Journal of Perinatal Education | 2 | No |
| Bergman | Perspect Health Inf Manag | 4 | No |
| Bhowmick | Int J Health Geogr | 4 | No |
| Birbeck | Am J Trop Med Hyg | 6 | No |
| Blixen | J Gen Intern Med | 4 | Yes |
| Borreani | BMC Oral Health | 10 | No |
| Bowling | Genetics | 2 | No |
| Boyington | Prev Chronic Dis | 7 | No |
| Braun | J Gen Intern Med | 8 | No |
| Brickley | AIDS Behav | 2 | No |
| Bryan | J Natl Med Assoc | 4 | No |
| Buckloh | Diabetes Care | 13 | No |
| Burgoyne | BMC Public Health | 6 | No |
| Burke | BMC Res Notes | Information missing | No |
| Burnet | J Gen Intern Med | 13 | Yes |
| Bwambale | BMC Public Health | 4 | No |
| Cabassa | Soc Sci Med | Information missing | No |
| Carpenter | Oncol Nurs Forum | Information missing | No |
| Castel | Qual Life Res | 4 | Yes |
| Chen | BMC Public Health | 1 | No |
| Chiang | Prev Chronic Dis | 6 | No |
| Christensen | BMC Med Inform Decis Mak | 3 | No |
| Clemensen | Int J Telemed Appl | 1 | No |
| Cockcroft | BMC Health Serv Res | 96 | No |
| Constantine | Prev Chronic Dis | 2 | No |
| Crawford | Health Qual Life Outcomes | 15 | No |
| Cremer | Medscape J Med | 11 | No |
| Davis | Obesity | 8 | Yes |
| de Boer | BMC Public Health | 4 | No |
| de Matos | J Poverty | 8 | No |
| de Morton | Health Qual Life Outcomes | 3 | No |
| DiIorio | Prev Chronic Dis | 1 | No |
| Djibuti | Hum Resour Health | 3 | No |
| Dodor | Ghana Med J | 16 | No |
| Donnelly | J R Soc Med | 4 | No |
| Dwyer | Int J Behav Nutr Phys Act | 9 | Yes |
| Eddy | Nurs Clin North Am | 4 | No |
| Egede | J Gen Intern Med | 9 | Yes |
| Elstad | Prev Chronic Dis | 7 | No |
| Eshrati | Harm Reduct J | 3 | No |
| Fernández-Dávil | Sexualities | 7 | Yes |
| Fitzsimons | BMC Public Health | Information missing | No |
| Fjeld | Int Breastfeed J | 9 | No |
| Folta | Prev Chronic Dis | 4 | No |
| Fongwa | Vasc Health Risk Manag | 5 | Yes |
| Forster | BMC Pregnancy Childbirth | 8 | No |
| Fried | J Am Geriatr Soc | 13 | Yes |
| Fu | BMC Psychiatry | 5 | No |
| Furler | BMC Health Serv Res | 4 | No |
| Garratt | Health Qual Life Outcomes | Information missing | No |
| Geller | Patient Educ Couns | 3 | No |
| Gigantesco | Clin Pract Epidemol Ment Healt | 2 | No |
| Goldstein | J Gen Intern Med | 4 | Yes |
| Grace | BMJ | 17 | No |
| Greeff | Afr J Nurs Midwifery | 43 | No |
| Greenhalgh | BMJ | 7 | No |
| Griffin | Health Promot Pract | 3 | Yes |
| Gupta | BMC Pediatr | 8 | No |
| Gutteling | BMC Gastroenterol | 3 | Yes |
| Hanson | BMC Public Health | Information missing | No |
| Haq | Int J Ment Health Syst | 6 | No |
| Harawa | Arch Sex Behav | 7 | No |
| Hassall | Vox Sang | 8 | No |
| Hassan | Malar J | 8 | No |
| Hatchette | BMC Pediatr | 5 | Yes |
| Helm | Prog Community Health Partners | 14 | No |
| Heritage | Reproductive Health | 2 | No |
| Hicks | J Gen Intern Med | 6 | No |
| Hollenbeck | Qual Life Res | Information missing | No |
| Horn | BMC Med Educ | 4 | Yes |
| Horne | Age Ageing | 15 | No |
| Hsu | Ann Fam Med | 9 | Yes |
| Hudson | BMC Palliat Care | 2 | No |
| Irimu | Afr Health Sci | 8 | No |
| Janke | Psychosomatics | 8 | Yes |
| Jaspan | Vaccine | 18 | No |
| Jerome | J Med Libr Assoc | 1 | No |
| Johnson | Tob Induc Dis | 5 | No |
| Jolly | Prev Chronic Dis | 3 | No |
| Joos | BMC Health Serv Res | 3 | Yes |
| Kaholokula | Patient Educ Couns | 4 | No |
| Kaye | BMC Public Health | 6 | Yes |
| Kerr | Alcohol Clin Exp Res | 2 | No |
| Kerr | Can Fam Physician | 9 | Yes |
| Kerrison | J R Soc Med | Information missing | No |
| Khowaja | Ther Clin Risk Manag | 6 | No |
| Konkle-Parker | J Assoc Nurses AIDS Care | 3 | No |
| Kothari | Implement Sci | 2 | No |
| Krohne | Br J Gen Pract | 4 | No |
| Kwan | BMC Complement Altern Med | 16 | No |
| Lachapelle | Pain Res Manag | 11 | No |
| Lane | Sex Transm Infect | 3 | No |
| Lapane | J Gen Intern Med | 64 | No |
| Lau | Can Fam Physician | 7 | No |
| Lee | Patient Educ Couns | Information missing | No |
| Lee | Nutr Res Pract | 3 | Yes |
| Leung | J Ethn Subst Abuse | 3 | No |
| Liddy | Can Fam Physician | 3 | No |
| Lie | Med Educ Online | 9 | No |
| Lin | J Gen Intern Med | 6 | No |
| Livaudais | J Immigr Minor Health | 3 | No |
| Lloyd | BMC Health Serv Res | 4 | No |
| Locke | J Gen Intern Med | Information missing | No |
| Lofgren | Cost Eff Resour Alloc | 4 | No |
| Long | BMC Health Serv Res | 12 | No |
| Lu | Can Fam Physician | 2 | No |
| Magin | Can Fam Physician | 4 | Yes |
| Mahendradhata | BMC Public Health | 4 | No |
| Makoae | Assoc Nurses AIDS Care | 43 | No |
| Masiye | Malawi Med J | 8 | No |
| Mathias | Health Qual Life Outcomes | 3 | No |
| McCarty | Am J Med Genet A | 14 | No |
| McEachan | BMC Public Health | 14 | No |
| McGuire | Genet Med | 3 | No |
| Medina | Am J Pharm Educ | 2 | No |
| Meyer | J Rural Health | 34 | No |
| Mikhael | J Gen Intern Med | Information missing | No |
| Mikkelsen | BMC Health Serv Res | Information missing | No |
| Moeke-Maxwell | Int J Ment Health Syst | 7 | No |
| Mohler | Curr Med Res Opin | 8 | Yes |
| Morrison-Beedy | J Obstet Gynecol Neonatal Nurs | 4 | No |
| Mühlbacher | Psychosoc Med | 3 | No |
| Mulvaney | Diabetes Educ | 6 | Yes |
| Murphy | Am J Bioeth | 16 | No |
| Nagle | BMC Health Serv Res | 4 | Yes |
| Nanyingi | J Ethnobiol Ethnomed | Information missing | No |
| Nedjat | BMC Med Educ | 5 | No |
| Neergaard | BMC Palliat Care | 3 | Yes |
| Newman | Vaccine | 15 | No |
| Ng'ang'a | Malar J | 8 | No |
| Nguyen | Hum Resour Health | 2 | No |
| Nicholas | Health Qual Life Outcomes | 1 | No |
| Nicolaidis | J Gen Intern Med | 5 | Yes |
| Nilsson | Open Nurs J | 5 | Yes |
| O'Brien | Health Qual Life Outcomes | 6 | Yes |
| O'Donnell | Br J Gen Pract | 6 | Yes |
| Panchanadeswaran | Int J Drug Policy | 8 | No |
| Pangarakis | Heart Lung | 3 | No |
| Paradies | Int J Equity Health | 3 | No |
| Pardeshi | Indian J Community Medicine | Information missing | No |
| Patro | Indian J Community Med | 60 | No |
| Patton | BMJ | 1 | No |
| Paul | AMIA Annu Symp Proc | 7 | No |
| Peek | J Gen Intern Med | 4 | Yes |
| Perez | BMC Public Health | 4 | No |
| Pettman | Int J Behav Nutr Phys Act | Information missing | No |
| Pfefferle | Qual Health Res | 87 | No |
| Plugge | Br J Gen Pract | 6 | No |
| Pool | Malar J | 44 | No |
| Pottie | Can Fam Physician | 4 | No |
| Power | BMC Musculoskelet Disord | 8 | Yes |
| Probandari | Glob Health Action | Information missing | No |
| Puaina | Cancer Detect Prev | 6 | No |
| Quave | J Ethnobiol Ethnomed | Information missing | No |
| Revicki | Health Qual Life Outcomes | 14 | No |
| Rheinländer | J Urban Health | 4 | No |
| Rice | Br J Gen Pract | 3 | No |
| Richter | Prev Chronic Dis | 16 | No |
| Ridde | BMC Health Serv Res | 4 | No |
| Rosen | J Am Geriatr Soc | 16 | No |
| Rouf | BMC Med Educ | 1 | No |
| Roy | Indian J Occup Environ Med | 1 | No |
| Ruth | Public Health Rep | 4 | No |
| Sagbakken | BMC Public Health | 2 | No |
| Samady | Public Health Nurs | 2 | No |
| Saulo | Malar J | 2 | No |
| Scammell | Soc Sci Med | 3 | No |
| Schwappach | BMC Health Serv Res | 2 | No |
| Scott | Int J Equity Health | 6 | No |
| Shea | J Gen Intern Med | 12 | No |
| Shuval | Prev Chronic Dis | 8 | Yes |
| Sivan | J Pediatr Nurs | 15 | No |
| Sommerseth | Patient Prefer Adherence | 3 | No |
| Sque | J R Soc Med | 3 | No |
| Stalmeijer | Adv Health Sci Educ Theory Pra | 3 | No |
| Stein | BMC Health Serv Res | 3 | No |
| Strong | J Am Diet Assoc | 2 | Yes |
| Tao | AMIA Annu Symp Proc | Information missing | No |
| Tarcea | Nucleic Acids Res | Information missing | No |
| Taylor | BMC Public Health | 5 | No |
| Taylor | J Community Health | 6 | No |
| Taylor | J Immigr Minor Health | 4 | Yes |
| Tentler | J Gen Intern Med | 6 | No |
| Torpey | PLoS ONE | 10 | No |
| van Dellen | BMC Public Health | 12 | No |
| Van Rompay | Hum Resour Health | 5 | No |
| Voils | Patient Prefer Adherence | 12 | No |
| Vue | J Nutr Educ Behav | 7 | No |
| Wagner | J Ethn Subst Abuse | 4 | No |
| Waiswa | BMC Pregnancy Childbirth | 10 | No |
| Walsh | Qual Life Res | 8 | No |
| Wan | Int J Clin Pract | 6 | Yes |
| Watson | BMC Health Serv Res | 5 | Yes |
| Weerasinghe | BMC Public Health | 12 | No |
| Wegner | Health Educ Res | Information missing | No |
| Wei | BMC Public Health | 16 | No |
| Weyrich | BMC Med Educ | 1 | No |
| Whittaker | J Med Internet Res | 4 | No |
| Wiener-Ogilvie | BMC Fam Pract | 2 | No |
| Wihlman | Int J Integr Care | 14 | No |
| Wild | J Appl Gerontol | 6 | No |
| Williams | Malar J | 12 | No |
| Wills | Clin Interv Aging | Information missing | No |
| Winickoff | Pediatrics | 8 | No |
| Wong | Healthc Policy | 11 | No |
| Woolf | BMJ | 5 | No |
| Yaffe | Can Fam Physician | 4 | No |
| Yanagawa | Int Arch Med | 1 | No |
| Yi | J Obstet Gynecol Neonatal Nurs | 3 | No |
| Zickmund | J Gen Intern Med | 10 | No |
